# Novel Insights into the Cardio-Protective Effects of FGF21 in Lean and Obese Rat Hearts

**DOI:** 10.1371/journal.pone.0087102

**Published:** 2014-02-03

**Authors:** Vanlata Patel, Raghu Adya, Jing Chen, Manjunath Ramanjaneya, Muhammad F. Bari, Sunil K. Bhudia, Edward W. Hillhouse, Bee K. Tan, Harpal S. Randeva

**Affiliations:** 1 Division of Metabolic & Vascular Health, Warwick Medical School, University of Warwick, Coventry, United Kingdom; 2 Department of Pathology, Dow International Medical College, Karachi, Pakistan; 3 Department of Cardiothoracic Surgery, UHCW NHS Trust, Coventry, United Kingdom; 4 Hamad Medical Corporation & Weill Cornell Medical School, Doha, Qatar; University of Torino, Italy

## Abstract

**Aims:**

Fibroblast growth factor 21 (FGF21) is a hepatic metabolic regulator with pleotropic actions. Its plasma concentrations are increased in obesity and diabetes; states associated with an increased incidence of cardiovascular disease. We therefore investigated the direct effect of FGF21 on cardio-protection in obese and lean hearts in response to ischemia.

**Methods and Results:**

FGF21, FGF21-receptor 1 (FGFR1) and beta-Klotho (βKlotho) were expressed in rodent, human hearts and primary rat cardiomyocytes. Cardiac FGF21 was expressed and secreted (real time RT-PCR/western blot and ELISA) in an autocrine-paracrine manner, in response to obesity and hypoxia, involving FGFR1-βKlotho components. Cardiac-FGF21 expression and secretion were increased in response to global ischemia. In contrast βKlotho was reduced in obese hearts. In isolated adult rat cardiomyocytes, FGF21 activated PI3K/Akt (phosphatidylinositol 3-kinase/Akt), ERK1/2(extracellular signal-regulated kinase) and AMPK (AMP-activated protein kinase) pathways. In Langendorff perfused rat [adult male wild-type wistar] hearts, FGF21 administration induced significant cardio-protection and restoration of function following global ischemia. Inhibition of PI3K/Akt, AMPK, ERK1/2 and ROR-α (retinoic-acid receptor alpha) pathway led to significant decrease of FGF21 induced cardio-protection and restoration of cardiac function in response to global ischemia. More importantly, this cardio-protective response induced by FGF21 was reduced in obesity, although the cardiac expression profiles and circulating FGF21 levels were increased.

**Conclusion:**

In an ex vivo Langendorff system, we show that FGF21 induced cardiac protection and restoration of cardiac function involving autocrine-paracrine pathways, with reduced effect in obesity. Collectively, our findings provide novel insights into FGF21-induced cardiac effects in obesity and ischemia.

## Introduction

The pandemic of obesity is associated with a critical increase in atherosclerotic cardiovascular disease (CVD) that is now one of the leading causes of global mortality and morbidity [1], specifically; atheromatous growth in the vascular wall causes life-threatening myocardial infarction (MI) [2]. Impairment of cardiac function following MI activates innate protective mechanisms that limit myocardial injury and promote repair [3]. These include increased production of cardiomyocyte survival factors from distant organs such as the liver and adipose tissue [4], [5]. Hepatic involvement in cardio-protection of experimental MI has been recently reported [6]. *In-vivo* administration of liver extract from donor mice with acute MI, rescued the injured myocardium, suggesting the presence of hepatic secreted cardioprotective factors [7].

The fibroblast growth factor (FGF) family is abundantly expressed in the liver and white adipose tissue (WAT) regulating multiple physiological functions including growth, development, angiogenesis and wound healing [8], [9]. Notably, the hepatokine/adipokine FGF21, circulating levels of which are elevated in obesity and type 2 diabetes [10], [11], has been implicated as a key metabolic regulator. In relation to this, the concept of ‘FGF21 resistance’ in obesity has been proposed [12], [13]. Furthermore, higher FGF21 levels were observed in dyslipidemic patients with coronary heart disease [14]. However, no study has shown the direct involvement of FGF21 in obesity related CVD. Therefore, we sought to elucidate the role of FGF21 in mediating myocardial protection following MI within this context.

## Methods

### Animals

#### Ethics statement

AWERB (Animal Welfare and Ethical Review Body) oversees all work with animals within the University (both general and project specific). This study did not go directly to the committee for approval as it technically did not involve any regulated procedure and therefore did not require a Home Office Project licence to cover the work. One of the authors (VP) involved in experimental design holds a Home Office personal licence for experimental work with animals. All animal technicians also hold personal licences. The University of Warwick, UK has an establishment licence that covers the facility and as such, any work conducted within it.

All animals were checked at least daily in compliance with the Animal Scientific Procedures Act 1986 (ASPA). Animals were also weighed and health checked weekly by dedicated independent animal technicians. Any rats showing abnormal weight gains or losses, or any rats showing initial signs of ill health as a result of the high fat diet (or any other related conditions), were culled immediately using a schedule 1 method.

For the experiments, rats were sacrificed by an intraperitoneal administration of sodium pentobarbital (200 mg/kg), following which the hearts were rapidly excised, immersed in ice-cold oxygenated Tyrodes solution.

Adult male wild-type wistar rats [Charles River labs, UK] were housed individually under pathogen-free conditions with controlled temperature and humidity, had free access to standard chow diet or high fat diet and water. Both diets were obtained from RMI-Dietex International Ltd., Essex, UK [**see Table S1**]. The physical and metabolic characteristics of standard chow diet fed and high fat fed rats are summarized in Table S2. 86 rats on standard chow diet (lean) and 30 on high fat diet (obese) were used.

### Drugs

The drugs used for the study were: rat recombinant FGF21 [R&D systems, UK], wortmannin (PI3K inhibitor), U1026 (MAPK inhibitor), Compound C (AMPK inhibitor), TO-901317 [ROR-α (Retinoic acid receptor-related receptor alpha) inhibitor]; TO-901317 (was initially intended as a therapeutic Liver X receptor (LXR) agonist in dementia [15], however recently, work by Uebanso T et al, have demonstrated its role in the regulation of FGF21 expression [16]) and collagenase (type I+protease type XIV). All these were purchased from Sigma-Aldrich and were of the highest purity available.

### Isolated Langendorff Preparation

The isolated heart Langendorff system as an *ex vivo* technique has unique advantages in reproducibility, low cost operability and measuring wide range of cardiac parameters. The cardiac effect of pharmacological agents can be studied in isolation, since the heart is devoid of bodily hormonal and nervous control, thereby making the Langendorff system ideal for investigating dose-response effects in metabolic and pharmacological experiments. However, potential disadvantages include time limitations and degradation of cardiac preparation in an *ex vivo* system, as opposed to an *in vitro* system.

After a week of habituation, rats were sacrificed by intraperitoneal administration of sodium pentobarbital (200 mg/kg), and hearts were rapidly excised, immersed in ice-cold oxygenated Tyrodes solution and immediately perfused *via* the aortic cannula in a modified Langendorff mode with Tyrode’s solution at 37°C. For details **see Supporting Information S1.**

### Determination of Infarction Following Ischaemia/Reperfusion in the Isolated Langendorff Perfused Rat Heart

As depicted below, following 30 mins stabilisation (t0), global ischemia was induced for 30 mins by cessation of tyrodes inflow, maintained at 37°C, followed by 120 mins reperfusion. Contractile parameters were measured throughout the procedure [**see Supporting Information S1**].

Following this, hearts were sliced into 2 mm thick transverse sections, incubated in 1% triphenyl-tetrazolium chloride in phosphate buffer (pH 7.4, 37°C) for 15 mins.

Briefly, following reperfusion, hearts were weighed, frozen, and cut into 2-mm-thick sections from apex to base. The sections were stained with triphenyl-tetrazolium chloride for infarct size determination, staining viable tissue red.

Sections were fixed in 4% formalin and traced onto a clear acetate sheet to determine infarct size by computer-assisted planimetry [17], [18]. The total volume from each slice of the heart was calculated by multiplication of each area by 2 mm, i.e., the thickness of the heart slice.

**Figure 1:**
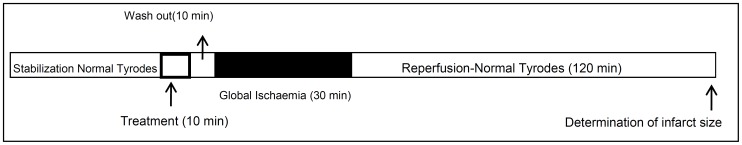
Experimental design. Following 30(t0), treatments were given for 10 mins followed by 10 minutes washout (normal tyrodes) and subsequently global ischemia was induced for 30 mins by cessation of tyrodes inflow, maintained at 37°C, followed by 120 mins reperfusion. Infarct size determination was performed subsequently.

Experimental groups are as follows- control (saline treated) lean/obese (n = 10 each), FGF21 treated lean/obese (n = 8 each), FGF21 treated with or without inhibitors [U1026/Compound C/TO-901317/wortmannin] (n = 6 each)], control vehicles treatments (n = 3 for each)]. Treatments were given for 10 mins followed of 10 minutes washout (normal tyrodes) and subsequently 30 mins global ischaemia and 120 mins reperfusion [**see** **Figure 1**].

### Primary Adult Rat Cardiomyocytes Isolation and Culture

In brief, isolated hearts were perfused on the Langendorff system with normal Tyrodes for 15 min (as above), followed by Ca^2+^-free Tyrode+collagenase type I+protease type XIV (Sigma) as described previously [19], [20][**see Supporting Information S1**].

### Immunohisto/Cytochemistry and Confocal Microscopy

Immunofluorescence was carried out in isolated cardiomyocyte cell suspension and immunohistochemistry was performed on formalin fixed paraffin embedded heart tissues [**see Supporting Information S1**].

### Detection and Measurement of Cardiac FGF21

Langendorff coronary effluent samples following ischemia and reperfusion were quantitatively analysed for the presence of secreted FGF21 protein. In brief, coronary effluents were collected, freeze-dried, and reconstituted with 0.5 mL PBS. Protein concentrations were quantified using a BCA-protein quantification kit as per manufacturer’s protocol (Pierce Biotechnology, USA) and equalized with PBS. FGF21 levels in concentrated exudates were measured using a commercially available ELISA (BioVendor, Oxford, U.K.), according to the manufacturer’s protocol.

### RNA Isolation and Real-time Quantitative Reverse Transcription Polymerase Chain Reaction

Total RNA was extracted using the RNeasy Mini Kit (Qiagen Ltd., UK) followed by reverse transcription into cDNA, as described previously, as was Q-RT- PCR [21] [**see Supporting Information S1**].

### Western Blot Analysis

Protein expression levels of FGF21 and its regulation in obese and ischemic rat hearts were measured by western blot analyses. Similarly, FGF21 induced activation of ERK_1/2_, Akt and AMPK in isolated rat cardiomyocytes were measured. Concentration (recombinant FGF21; 0–100 nM) and time (0–60 mins) dependent optimisation experiments were performed [**see Supporting Information S1**].

### Statistical Analysis

Differences between two groups were assessed using the unpaired t test (GraphPad Prism 5; GraphPad Software, San Diego, USA). Data involving more than two groups were assessed by ANOVA with post-hoc analysis: Dunnett (GraphPad Prism 5; GraphPad Software, San Diego, USA). Data are mean ± SEM. *P*<0.05 was considered significant.

## Results

### Identification of FGF21mRNA, Protein and the Effect of Recombinant FGF21 on Cardiac Function and Infarct Size

**Figure 2:**
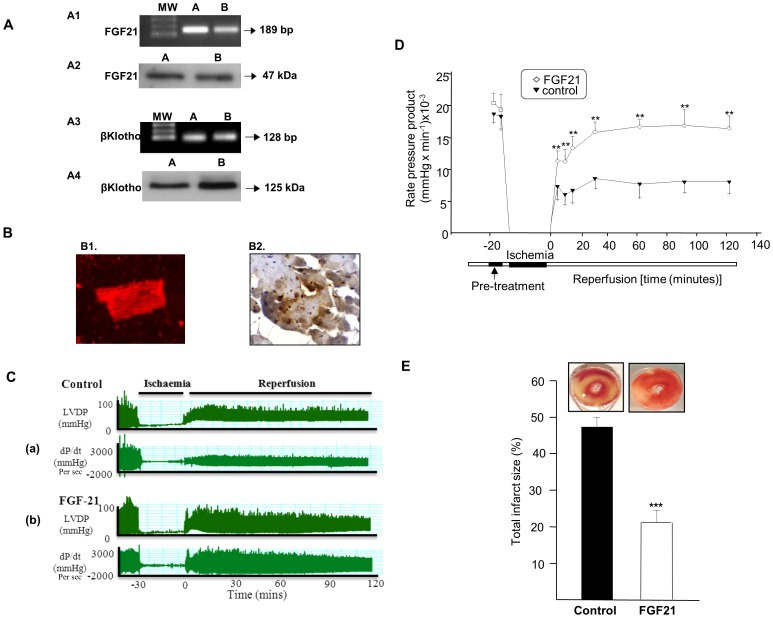
Identification of FGF21mRNA, protein and the effect of recombinant FGF21 on cardiac function and infarct size. FGF21 mRNA [**Fig. 2A1**] and protein expression [**Fig. 2A2**]; βKlotho mRNA [**Fig. 2A3**] and protein expression [**Fig. 2A4**] in isolated rat cardiomyocytes [A] and rat heart [B]. Fig**.** 1B:Immunocyto/histochemistry and confocal analysis of FGF21 protein in isolated adult rat cardiomyocytes [**B1**] and rat heart [**B2**]. MW.- Molecular Weight Marker for PCR products; BP.- Base Pairs; kDa.-kilo daltons. **Fig. 2C**-(**a**): Trace recordings of left ventricular developed pressure [LVDP (mmHg)] and left ventricular contractility (dp/dt) in control (saline treated) groups; and **Fig. 2C**-(**b**):[LVDP-mmHg] and dp/dt ratio in FGF21 treated groups - following 30 mins of global ischemia and 120 mins reperfusion. **Fig. 2D:** Rate pressure product [RPP (mmHg/min)] during global ischemia and reperfusion with or without FGF21 treatment [**P<0.01 vs. control]. **Fig. 2E**: Graphical representation of infarct area (%) in rat hearts treated with or without FGF21. Data shown are means ± SEM (n = 6, in triplicates). ***P<0.001; **P<0.01 vs. control.

RT-PCR analysis showed expression of FGF21 mRNA [**Fig. 2A** **1]** and western blot analysis showed corresponding protein levels [**Fig. 2A** **2**.] in adult rat cardiomyocyte [**A**], and [**B**] rat heart. Immunocyto/histochemistry and confocal analysis [**Fig. 2B**] showed intracellular FGF21 staining in adult rat cardiomyocyte [**B1**] and [**B2**] rat heart. Furthermore, we detected the FGF21 signalling component [cofactor essential for FGF21 activity [**22**], βKlotho mRNA [**Fig. 2A** **3**] and protein expression [**Fig. 2A** **4**] for the first time in adult rat cardiomyocyte [**A**] and [**B**] rat heart.

### Cardiac Function

In the isolated rat heart Langendorff system, we measured left ventricular developed pressure (LVDP), relating to maximal rates of LV pressure decay; dP/dtmin and dP/dtmax (+/−dp/dt), a specific indicator of iso-volumetric phase index of left ventricular contractility, respectively. **Fig. 2C** represents a real time data trace recording of both LVDP and (+/−dp/dt). Panel (**a**) demonstrates that the control (lean) group (saline treated), showed poor cardiac functional recovery and a significant decline in cardiac function following 30 mins global ischemia and 120 mins reperfusion. Control/lean hearts pre-treated with recombinant rat FGF21 (100 nM) showed significant improvement in functional recovery **(b**). Graphical representation pattern of rate pressure product (RPP) during global ischemia and reperfusion demonstrates pre-treatment with FGF21, resulting in significantly improved functional recovery [*P*<0.01*;* **Fig. 2D**].

### Infarct Size

Following global ischemia and reperfusion, with or without FGF21 pre-treatment, infarct size was determined using TTC staining for the control/lean group. Transverse sections of rat hearts demonstrated an increase in the viable area in those treated with FGF21, when compared with control group which showed a larger infarct area. Infarcted areas were assessed using computer assisted planimetry (NIH image 1.57). FGF21 pre-treatment, 10 mins prior to global ischemia, significantly reduced infarct size [*P*<0.001; **Fig. 2E**].

### The Role of FGF21 Activated Signalling Pathways in Cardioprotection

**Figure 3:**
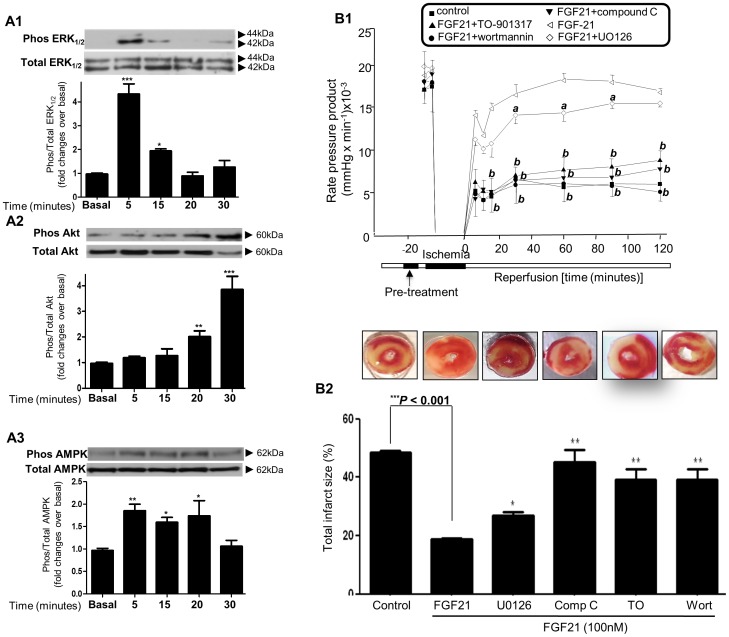
The role of FGF21 activated signalling pathways in cardioprotection. Fig. 3A1: Cardiomyocytes treated with FGF21 (100 nM) for 5–30 minutes. Phosphorylated ERK_1/2_ [Fig. 3A1]; Akt [Fig. 3A2] and AMPK [Fig. 3A3] proteins are represented in relation to total proteins, expressed as fold increase over basal. ***P<0.001, **P<0.01, *P<0.05 vs. basal, n = 6 per group. Fig. 3B1: RPP with ischemia/reperfusion and FGF21 treatment following pre-incubation with inhibitors (TO-901317; wortmanin; Compound C or U0126) ^a^P<0.05, ^b^P<0.01 vs. FGF21 only treatment, n = 6 per group. Fig. 3B2: Infarcted area (%) following ischemia/reperfusion and FGF21 treatment following pre-incubation with inhibitors (TO-901317; wortmanin; Compound C or U0126) **P<0.01, *P<0.05 vs. FGF21 treated, n = 6 per group.

Activation of cell survival pathways including MAPK, PI3k/Akt and AMPK play a critical role in conferring myocardial protection following MI [23], [24]. We studied the effect of FGF21 on these pathways in isolated adult rat cardiomyocytes. **Fig. 3A** **1–A3** demonstrates pathway activation of ERK_1/2,_ Akt and AMPK, respectively. Phosphorylation of ERK_1/2_ was significantly induced by FGF21 (100 nM) at 5 mins and 15 mins [*P*<0.001and *P*<0.05 respectively; **Fig. 3A** **1**]. FGF21 induced significant increase in Akt phosphorylation at 20 and 30 mins [*P*<0.01 and *P*<0.001 respectively; **Fig. 3A** **2**], and AMPK activation at 5, 15 and 20 mins [*P*<0.001, *P*<0.01 and *P*<0.05 respectively; **Fig. 3A** **3**].

To study the involvement of these in FGF21 induced cardio-protection, we employed small molecule inhibitors of ERK_1/2_, PI3K/Akt and AMPK pathways namely U0126, Wortmannin and Compound C. Additionally, since the canonical ROR (Retinoic acid receptor-related receptor) response element identified in the proximal promoter of the FGF21 gene critically influences FGF21 effects, we employed TO-901317 a small molecule inhibitor of ROR-α/FGF21 signalling pathway [**25**].

As shown in **Fig. 3B** **1**, FGF21 induced a significant improvement in cardiac function following ischemia/reperfusion. Rate pressure product (RPP) was significantly affected when perfused along with either wortmannin (10µM; *P*<0.01) or TO-901317 (*P*<0.01), Compound C (10µM; *P*<0.01), and less so with U0126 (10µM; *P*<0.05).

**Figure 3B** **2** denotes graphical representation of infarct size; FGF21 administered alone reduced infarct size to 18.8±0.51%, but in combination with Wortmannin, TO-901317 or compound C this protective effect decreased (45.1±4%; 44.9±3.8%, 34±4%; *P*<0.001, **Figure 3B** **2**) but not to the same degree with U0126 (26.8±2.3%; *P*<0.05), suggesting the involvement of these pathways. Additionally, these small molecule inhibitors and their carrier compounds treated individually showed no significant differences in either cardiac function or the infarct size (data not shown).

### Autocrine/Paracrine Effects of FGF21 in Rat Cardiomyocytes

Emerging therapeutic strategies involving autocrine/paracrine mechanisms mediated by growth factors released from cardiomyocytes have been implicated to play an essential role in the reparative process of the compromised heart [26].

**Figure 4:**
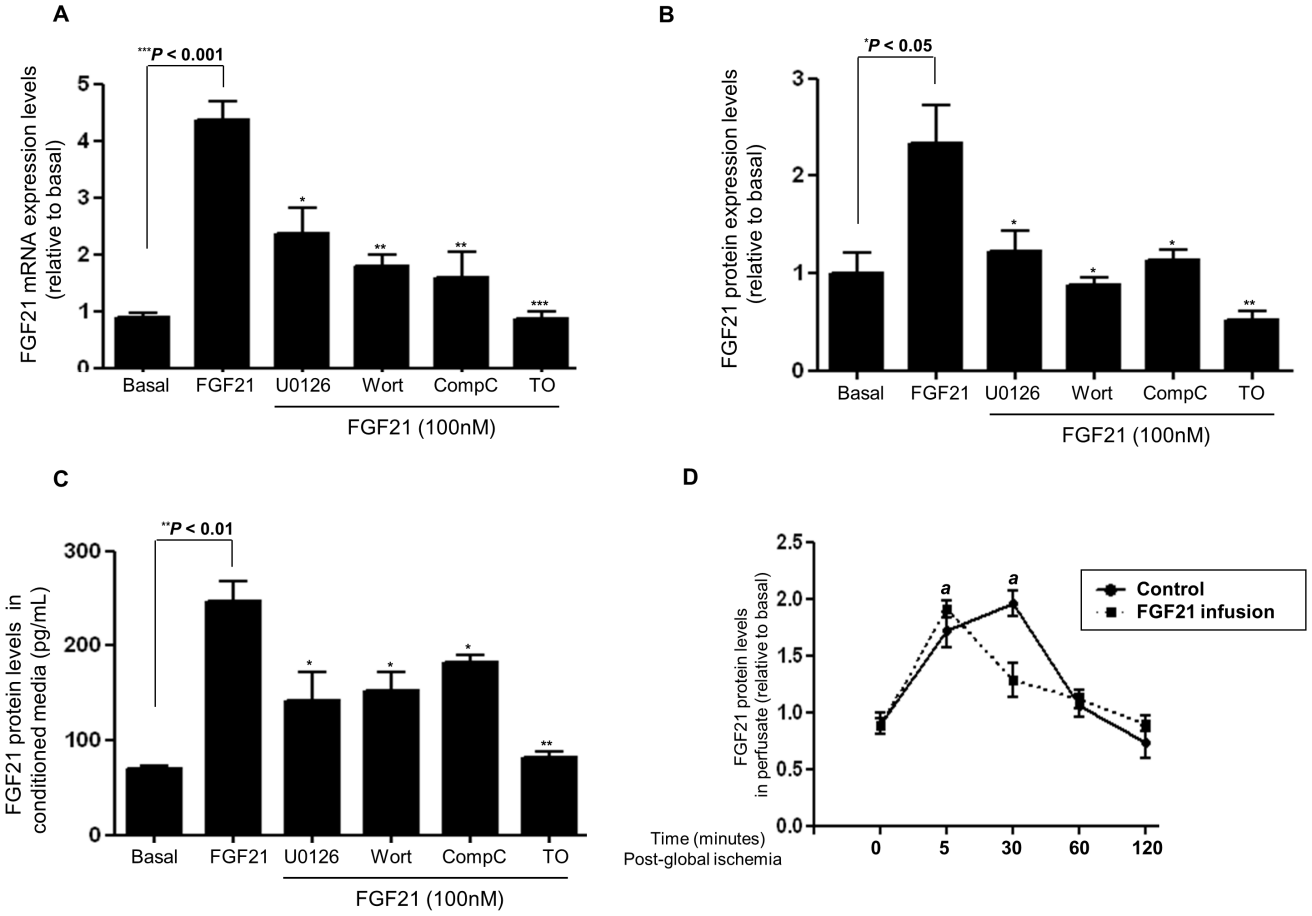
Autocrine/paracrine effects of FGF21 in rat cardiomyocytes. **Fig. 4A**:FGF21 mRNA expression levels in cardiomyocytes following FGF21 (100 nM) treatment with or without pathway inhibitors [(U0126; wort (wortmanin); Comp C (Compound C) or TO (TO-901317)] (normalised to GAPDH and expressed as fold changes over basal).**Fig. 4B**: Graphical analysis of FGF21 protein levels following FGF21 treatment. **Fig. 4C**: Graphical representation of FGF21 ELISA measurements in the conditioned media following FGF21 treatment. **Fig. 4D**: Graphical representation of FGF21 ELISA measurements of rat heart Langendorff exudates following global ischemia for 5–30 mins and 120 mins of reperfusion; with or without prior FGF21 (100 nM) infusion. Data shown are means ± SEM of triplicates. The values represented are relative to basal. ***P<0.001; **P<0.01; *P<0.05 vs. FGF21 only treated, **^a^** *P*<0.001 vs. basal, n = 6 per group.

Adult rat cardiomyocytes treated with recombinant rat FGF21 increased FGF21 mRNA with maximum response at 100 nM and 4 hours [*P*<0.001 vs. Basal; **Figure 4A**]. This was abrogated when cardiomyocytes were pre-incubated (for 60 minutes) with either wortmannin (10µM) [*P*<0.01] or TO-901317(10µM) [*P*<0.001] or Comp C (10µM) [*P*<0.01] or U0126 (10µM) [*P*<0.05] vs. FGF21 only treated. This was associated with a concomitant increase in FGF21 protein expression levels [*P*<0.05 vs. Basal; **Figure 4B**] and secretion into conditioned media [*P*<0.01 vs. Basal; **Figure 4C**].

In the Langendorff system, following recombinant rat FGF21 (100 nM) perfusion, induction of global ischemia and reperfusion, FGF21 protein levels were measured in Langendorff exudates at various time points. There was a maximum increase in cardiac secreted FGF21 levels following 30 mins global ischemia, and returning to basal levels following reperfusion after 120 mins [P<0.001 30 mins ischemia vs. Basal; **Figure 4D**]. However, this maximum increase in secreted FGF21 levels was observed much earlier at 5 mins following exogenous recombinant FGF21 pre-infusion, again implicating autocrine mechanisms. [P<0.001; 5 mins ischemia vs. Basal; **Figure 4D**]. Therefore, we postulate the existence of preformed FGF21 protein secretory granules which could account for the acute release of FGF21 from the heart following global ischemia.

Cardiac FGF21/FGFR1 expression levels and FGF21 secretion in response to global ischemia in controls/lean and obese rat hearts: reduction in βKlotho and MAPK/PI3k-Akt signalling in obese hearts.

#### a) Effects of global ischemia

**Figure 5:**
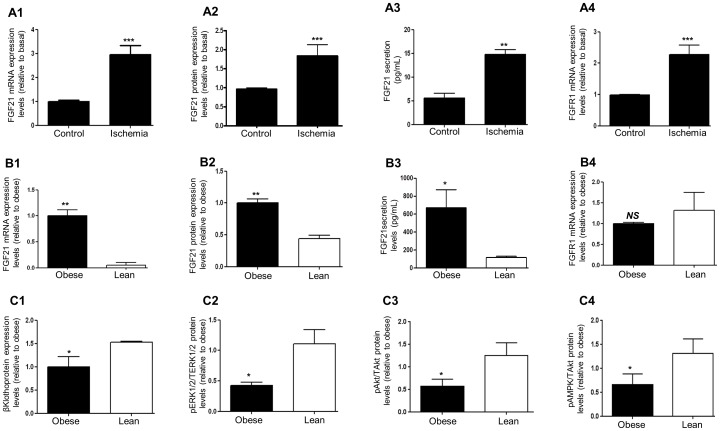
Cardiac FGF21/FGFR1 expression levels in secretion of FGF21 in response to ischemia and obesity: reduction in βKlotho and MAPK/PI3k-Akt signalling in obese hearts. Using rat heart Langendorff model and inducing global ischemia for 30[**Fig. 5A1**], protein [**Fig. 5A2**], and secretion of FGF21 in Langendorff coronary exudates [**Fig. 5A3**] were measured. Similarly, changes in FGFR1 mRNA expressions [**Fig. 5A4**] were measured. Data shown are means ± SEM of triplicates. The values represented are relative to basal. ***P<0.001; **P<0.01vs. control. n = 6 per group. FGF21 mRNA [**Fig. 5B1**], protein [**Fig. 5B2**], FGF21 secretion (in Langendorff coronary exudates) [**Fig. 5B3**] and FGFR1 mRNA [**Fig. 5B4**] expressions were measured in obese and lean rat hearts. Graphical representation of a key signalling component of FGF21/FGFR1; βKlotho protein level in obese and lean hearts [*P*<0.05 vs.lean; **Figure 5C** **1**]. Graphical representation of ERK_1/2_ [**Fig. 5C2**], Akt [**Fig. 5C3**] and AMPK [**Fig. 5C4**] phosphorylation levels in lean and obese hearts with FGF21 (100 nM) pre-infusion. Data shown are means ± SEM of triplicates. The values represented are relative to basal. ^*^ *P*<0.05, ^**^ *P*<0.01 vs. lean control, *NS*-non-significant; n = 6 per group.

Cardiac ischemia results in the activation of compensatory pro-survival pathways. We were interested to decipher the mechanisms of FGF21 induced myocardial protection using pathophysiological models of cardiac ischemia in lean and obese states. In an isolated rat heart Langendorff model, following global ischemia for 30 mins there was a significant increase in cardiac FGF21 mRNA, protein and secretion levels [*P*<0.001, *P*<0.01 global ischemia vs. Basal; Figure 5A1–A3]. There was also a concurrent rise in FGFR1mRNA, a key signalling receptor for FGF21 following global ischemia [*P*<0.001; Figure 5A4].

#### b) Effects in obesity

We describe for the first time, relative differences in lean vs. obese rat cardiac expressions of FGF21 and its signalling components FGFR1 and βKlotho. We observed a significant increase in FGF21 mRNA, FGF21 protein and FGF21 secretion in obese rat hearts [*P*<0.01; *P*<0.05 vs.lean; Figure 5B1–B3]. Cardiac FGFR1 mRNA expression levels were non-significant between control lean and obese groups [*P* = *NS*; Figure 5B4]. Both βKlotho (an essential co-factor for FGF21 signalling) mRNA (data not shown) and protein levels was significantly decreased in obese hearts [*P*<0.05 vs.lean; Figure 5C1], further supporting the concept of decreased FGF21/FGF-R1/βKlotho signalling in obesity. To elucidate the relevance of this finding we further investigated the effect of FGF21 treatment on ERK_1/2_, Akt and AMPK phosphorylation levels. ERK_1/2_ phosphorylation was significantly decreased in obese cardiac tissue following FGF21 infusion (100 nM) using the Langendorff model [*P*<0.05; Figure 5C2]. Similar findings were demonstrated in the case of Akt [*P*<0.05; Figure 5C3] and AMPK phosphorylation states [*P*<0.05; Figure 5C4], further supporting decreased FGF21 signalling in cardiac tissue in obesity.

### Effect of Global Ischemia on Cardiac Function, Infarct Size in Obese Rat Heart: differences in FGF21 Autocrine Secretory Pattern

#### Cardiac function

**Figure 6:**
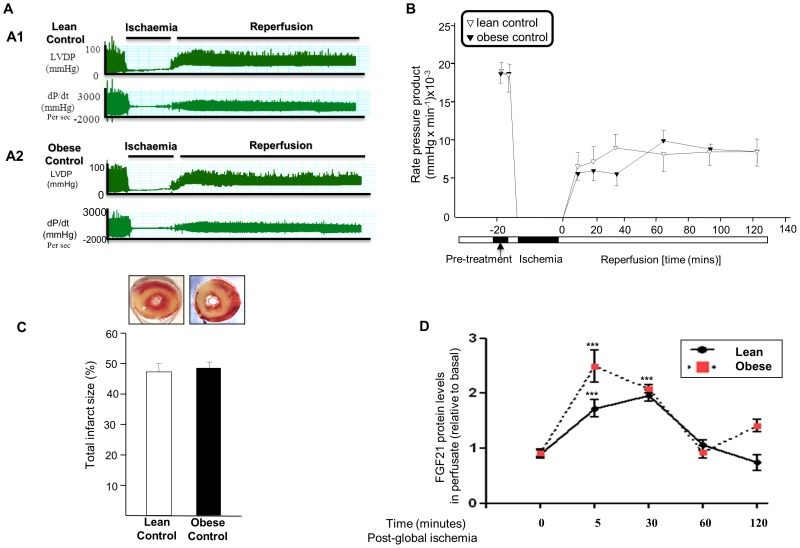
Effect of global ischemia on Cardiac Function, Infarct Size in Obese Rat Heart: differences in FGF21 autocrine secretory pattern. LVDP (mmHg) and left ventricular contractility (dp/dt) in obese control (saline treated) groups [**Fig. 6A1**] and lean control (saline treated) groups [**Fig. 6A2**]; following global ischemia and reperfusion. Graphical representation of RPP (mmHg/min)] during global ischemia and reperfusion in obese and lean control rat hearts [**Fig. 6B**]. Graphical representation of the infarcted area (%) in obese and lean control rat hearts following global ischemia and reperfusion [**Fig. 6C**]. Graphical representation of FGF21 levels in obese and lean rat heart coronary effluents following global ischemia and reperfusion [**Fig. 6D**]. Data shown are means ± SEM of triplicates. The values represented are relative to basal.^ ***^ *P*<0.001 vs. t [0] time point, n = 6 per group.

As mentioned above, to study these differences in obese and lean hearts, we examined the effect of global ischemia in an isolated rat heart Langendorff model. We measured LVDP, +/−dp/dt and APP in both lean and obese rat hearts [saline and FGF21 (100 nM) treated]. As represented in Fig**.** 6A, a real time data trace recording of LVDP+/−dp/dt in lean saline treated groups (Fig**.** 6A1); and obese saline treated groups (Fig**.** 6A2). Fig**.** 6B denotes graphical representation pattern of RPP during global ischemia and reperfusion in obese and lean saline perfused hearts. As depicted, there was no significant changes in RPP between lean and obese saline treated hearts (following 30 mins global ischemia) [P = NS; Fig**.** 6B].

#### Infarct size

Following global ischemia and subsequent reperfusion for 120 mins (10 mins prior to 30 mins global ischemia) in obese and lean hearts, infarct size measurements were performed. There was no significant differences in the total area of infarct between lean and obese saline treated hearts) [P = NS; Fig**.** 6C].

#### FGF21 Secretion in lean and obese langendorff preparations

As mentioned previously following global ischemia for 30 mins, FGF21 protein levels were measured in Langendorff exudates at various time points following reperfusion in lean and obese hearts. In lean hearts, there was a maximum increase in cardiac secreted FGF21 levels following 30 mins reperfusion (post ischemia) [P<0.001 30 mins *vs* 0 min reperfusion; Figure 6D]. However, in obese heart Langendorff exudates, maximum FGF21 protein levels were observed at 5 mins of reperfusion [*P*<0.001 5 mins vs 0 min reperfusion; Figure 6D]. As observed in Figure 5B3, we noted that the amount of FGF21 produced (area under the curve) by obese hearts was significantly higher compared to the lean counterparts (*P*<0.01).

### Differences in Cardiac Function, Infarct Size and FGF21 Autocrine Secretory Pattern in Lean and Obese Hearts Treated with FGF21

Similar experiments were conducted in lean and obese hearts but with FGF21 (100 nM) infusion prior to the induction of global ischaemia.

#### Cardiac function

**Figure 7:**
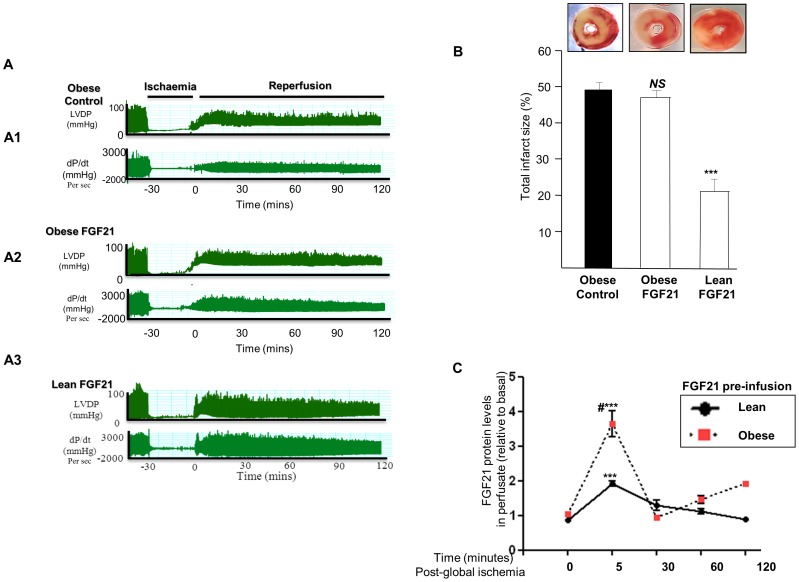
Differences in Cardiac Function, Infarct Size and FGF21 autocrine secretory pattern in lean and obese hearts treated with FGF21. LVDP (mmHg) and left ventricular contractility (dp/dt) in obese control (saline treated) groups [**Fig. 7A1**], obese (FGF21 treated) groups [**Fig. 7A2**] and lean (FGF21 treated) groups [**Fig. 7A3**]; following global ischemia and reperfusion. Graphical representation of the infarcted area (%) in obese control, obese and lean FGF21 (100 nM) treated rat hearts following global ischemia and reperfusion [**Fig. 7B**]. Graphical representation of FGF21 levels in obese and lean FGF21 (100nM) pre-treated rat heart coronary effluents following global ischemia and reperfusion [**Fig 6C** **]**. Data shown are means ± SEM of triplicates. The values represented are relative to basal. ^***^ *P* < 0.001 vs. t [0] time point, **^#^** *P* < 0.05 vs. lean at t(5); n  =  6 per group.

Fig**.** 7A represents a real time data trace recording of both LVDP and (+/−dp/dt), in (A1) obese group (saline treated), (A2) obese group (FGF21treated) and (A3) lean group (FGF21 treated).

#### Infarct size

Infarct size measurements were performed in lean and obese hearts treated with FGF21 (100 nM), following global ischemia and subsequent reperfusion for 120 mins. There was no significant differences in the total area of infarct between obese saline treated hearts and obese FGF21 treated hearts (P = NS; Fig**.** 7B). However as mentioned previously, infarct size was significantly reduced in lean FGF21 treated hearts compared to their saline treated counterparts [*P*<0.001 vs lean saline treated hearts; Fig**.** 7B].

#### FGF21 Secretion in lean and obese langendorff preparations following FGF21 pre-infusion

Obese and lean heart Langendorff preparations were pre infused with FGF21 (100 nM) and were subjected to global ischemia for 30 minutes. FGF21 protein levels were measured in Langendorff exudates at various time points following reperfusion. In both lean and obese heart perfusates, there was a maximum increase in cardiac secreted FGF21 levels following 5 mins reperfusion (post ischemia) [P<0.001 30 mins vs 0 min reperfusion; Figure 7C]. However, there was a significant increase in FGF21 levels secreted from the obese heart in comparison with the lean ones [*P*<0.001 Figure 7C].

## Discussion

Our data provides novel insights into the cardio-protective effects of FGF21 within the context of obesity related CVD. Specifically, we show for the first time that FGF21 infusion into a Langendorff perfused rat heart significantly confers myocardial protection and revival of cardiac function following MI. This effect appears to be mediated by the key cardiac cell survival pathways i.e. ERK_1/2_ (apparent immediate effect), AMPK (apparent immediate effect) and PI3K/Akt (apparent sustained effect) as well as ROR-α.

Importantly, we demonstrate that FGF21 is secreted by cardiomyocytes; thus, FGF21 is a novel cardiomyokine. Moreover, we found that FGF21 stimulates FGF21 production and secretion from cardiomyocytes. This translates into an autocrine/paracrine ‘positive feedback’ loop effect of FGF21 on cardiomyocytes, which further promotes the cardio-protective effects on the heart; this is similar to adiponectin, another notable hepatokine/adipokine [27]. In relation to this, recent studies have described the autocrine/paracrine actions of cardiomyokines released during myocardial injury [28]. Also, this autocrine/paracrine ‘positive feedback’ loop appears to be mediated by the ERK_1/2_, AMPK, PI3K/Akt signalling pathways and ROR-α. A disruption of these adipokine signalling mechanisms leads to a deranged autocrine/paracrine feedback loop; these feedback loops are very well recognised in obesity and diabetes mellitus [29]. We also show that FGF21 production and secretion was significantly higher in hearts subjected to acute global ischemia. Once again, this supports our findings on the cardio-protective effects of FGF21 with respect to MI. Our findings compliment the recent report by Planavila *et al*, that FGF21 is involved in protection against cardiac hypertrophy [30].

To further elaborate on the involvement of multiple signalling cascades in FGF21 induced cardiac protection; experimental observations have implicated the activation of PI3K-Akt and MAPK (ERK_1/2_) signalling pathways conferring myocardial protection [31]. In line with this, FGF21 induced myocardial protection involves the participation of these pathways albeit to different extents. Notably the degree of cardiac protection induced by FGF21 was significantly less when ERK_1/2_ was inhibited in comparison with PI3k/Akt, indicating the later to be a predominant pathway. We postulate that the role of ERK_1/2_ may be minimal *in vivo* and that cardiac actions of FGF21 are more selective towards PI3K/Akt signalling. However there is a possibility of existence of cross-talk between these pathways. As supported by recent findings, the inter-linking of ERK_1/2_ and PI3K/Akt networks facilitates linear signalling conduits activated by different stimuli [32].

Obesity is associated with a significantly higher risk of MI; this fact is supported by a powerful report by Yusuf et al. which examined a total of 27,098 participants from 52 countries, enlightening obesity to be an independent risk factor when known risk factors had been accounted for [32]. Indeed, it is well known that overweight and obese individuals have a significantly higher risk of developing coronary artery disease and some studies suggest that obese subjects fare poorly after a myocardial insult with higher morbidity and mortality [33], [34]; equally there are numerous studies reporting that obese individuals have a better outcome following MI which has resulted in the *obesity paradox* [35]. It is important to bear in mind that obese subjects are defined solely by BMI without considering other metabolic parameters such as circulating cholesterol and triglycerides. In rodents, a study by Thim et al reported no differences in infract size between lean and DIO rat hearts following MI [36]. Our findings are by and large consistent with their report.

In our diet induced obese (DIO) rat model we found that obese rats had significantly higher cardiac FGF21 mRNA and protein, as well as circulating FGF21 levels compared to lean rats; of relevance, higher circulating levels of FGF21 have been found in obese humans [11]. Interestingly, FGF21 infusion did not improve cardiac function and/or reduce infarct size in obese rat hearts following global ischemia, despite increased production and secretion of FGF21 in these obese rat hearts. In order to explain this paradox, we sought to explore the involvement of alternative signalling pathways.

The FGF21-FGFR1-βKlotho signalling pathway has been recognised as a novel therapeutic target in enhancing FGF21 action on target organs [37]. Genetic studies have demonstrated FGFR1 as a novel susceptibility gene in human obesity with increased adipose tissue levels in obese compared to lean subjects [38]. Interestingly, we found no significant difference in FGFR1 levels between obese and lean rat hearts. Importantly, βKlotho [signalling co-factor functioning as a cell surface adaptor molecule binding to C-terminus of FGF21 component of FGF21/FGFR1 signalling complex] [39] has been proposed to modulate actions of FGF21 [40]. Moreover, studies have suggested decreased hepatic/adipose tissue expression levels of βKlotho in human obesity and DIO rodents resulting in decreased FGF21 signalling in target organs [12], [41]. Hence, given our novel observation of βKlotho in the heart and a significant decrease in cardiac gene and protein expression levels of βKlotho in obese rat hearts, may account for the ‘FGF21 resistance’ observed in our *ex vivo* experiments, which in turn disrupts FGFR1-FGF21-βKlotho signalling. Furthermore, disruption of this signalling pathway in turn leads to attenuated ERK_1/2_, Akt and AMPK phosphorylation in obese compared to lean rat hearts, which may explain the ‘FGF21 resistance’ observed in obese rat hearts.

FGF21 has been widely studied as a metabolic molecule, up regulating glucose uptake in adipocytes *via* the AMPK pathway [10]. Our experimental results indicate the activation and involvement of AMPK in FGF21 induced cardiac protection possibly facilitating increased glucose uptake in ischemic stress [42].

The differences in glucose uptake have been noted in obese and lean hearts [43], which may account for the differences in FGF21 induced cardiac protection. However detailed studies need to be performed to elucidate the action of FGF21 in the heart.

Given that our novel observations demonstrate the direct effect of FGF21 in the heart and differences in obesity, a detailed analysis of the FGFR1-FGF21-βKlotho signalling pathway is crucial. Additionally, pharmacological/small molecule inhibitors have been scrutinised to be occasionally non-specific, i.e. simultaneous inhibition of unrelated pathways etc., caution needs to be exercised in evaluating the specific involvement of these pathways in FGF21 inducted effects. Specific cardiac gene silencing approaches are necessary to clarify the involvement of individual signalling components. Furthermore, differences in cardiac FGF21 signalling in human subjects needs to be considered. This was beyond the scope of the current study but requires attention by investigators in the future.

Additionally, the cardiac tissue release of proteins and substrates into the perfusate has been controversial. There are studies indicated that only irreversible injured cardiomyocytes can release cardiac proteins into the perfusate, whilst more recent studies have indicated physiological permeability of the cell membrane as a metabolically dependent process and extracellular rise of myocardial proteins in smaller amounts may occur in reversible disturbances of cell metabolism as well. Interestingly we that found that the release of FGF21 by the ischaemic Langendorff heart follows a divergent pattern in comparison with the release of cardiac troponin-T (cTn-T), a marker of cardiomyocyte cell death [**see Supporting Information S1**] [44].

Isolated Langendorff systems have been implicated in an artificially increased washout of interstitial proteins not reflecting in vivo findings, hence the *ex vivo* results need to be carefully interpreted [45].

In conclusion, we demonstrate the cardiac FGFR1-FGF21-βKlotho system and cardio-protective effects of FGF21. Our findings also highlight an autocrine/paracrine ‘positive feedback’ loop effect of FGF21 on cardiomyocytes leading to elevated cardiac FGF21 production and secretion. This, in turn, further exerts FGF21 mediated cardio-protective effects on the heart. Our novel data also shows the functionally significant differences in cardiac secreted FGF21 in obesity involving βKlotho. Finally, our data add to the diverse effects of FGF21, but more importantly reveal novel insights into the potential role(s) of FGF21 in human CVD as well as introduces novel translational perspectives. Also, given the paucity of effective clinical interventions for CVDs, we hope that our data will stimulate further efforts into this area of research.

## Supporting Information

Figure S1 **Comparison between FGF21 and cTn-T release in Langendorff perfusates.** Graphical representation of FGF21 and cTn-T (relative to basal) from Langendorff rat heart following global ischemia and reperfusion (5, 30, 60 and 120 minutes). Data shown are means ± SEM of triplicates. The values represented are relative to basal. ***P<0.001 vs. t [0] time point (FGF21), #*P*<0.05 vs. t [0] (cTn-T); n = 6 per group.
(DOCX)

Table S1 **Constituents of standard chow diet and high fat diet.**
(DOCX)

Table S2 **Physical and metabolic profiles in lean and high fed fat rats following 12 weeks of either standard chow diet (lean rats) or high fat diet (HF rat).**
(DOCX)

Table S3 **Primer sequences.**
(DOCX)

Table S4 **Haemodynamic parameters of isolated rat hearts.**
(DOCX)

Supporting Information S1 **Supporting Materials and Methods, and Results.**
(DOC)

## References

[1] Braunwald E (1997) Shattuck lecture–cardiovascular medicine at the turn of the millennium: triumphs, concerns, and opportunities. N Engl J Med 337: 1360–1369. 9358131 10.1056/NEJM199711063371906

[2] Shah PK, Forrester JS (1991) Pathophysiology of acute coronary syndromes. Am J Cardiol 68: 16C–23C. 10.1016/0002-9149(91)90219-b 1951098

[3] Timmers L, Pasterkamp G, de Hoog VC, Arslan F, Appelman Y, et al (2012) The innate immune response in reperfused myocardium. Cardiovasc Res 94: 276–283. 22266751 10.1093/cvr/cvs018

[4] Liu SQ, Tefft BJ, Liu C, Zhang B, Wu YH. (2011) Regulation of hepatic cell mobilization in experimental myocardial ischemia. Cell Mol Bioeng 4: 693–707, 2011.

[5] Valina C, Pinkernell K, Song YH, Bai X, Sadat S, et al (2007) Intracoronary administration of autologous adipose tissue-derived stem cells improves left ventricular function, perfusion, and remodelling after acute myocardial infarction. Eur Heart J 28: 2667–2677. 17933755 10.1093/eurheartj/ehm426

[6] Liu SQ, Tefft BJ, Roberts DT, Zhang LQ, Ren Y, et al (2012) Cardioprotective proteins upregulated in the liver in response to experimental myocardial ischemia. Am J Physiol Heart Circ Physiol 303: H1446–1458. 23064833 10.1152/ajpheart.00362.2012

[7] Liu SQ, Wu YH (2010) Liver cell-mediated alleviation of acute ischemic myocardial injury. Front Biosci (Elite Ed) 2: 711–724. 20036915 10.2741/e131

[8] Nishimura T, Nakatake Y, Konishi M, Itoh N (2000) Identification of a novel FGF, FGF-21, preferentially expressed in the liver. Biochim Biophys Acta 1492: 203–206. 10858549 10.1016/s0167-4781(00)00067-1

[9] Beenken A, Mohammadi M (2009) The FGF family: biology, pathophysiology and therapy. Nat Rev Drug Discov 8: 235–253. 19247306 10.1038/nrd2792 PMC3684054

[10] Kharitonenkov A, Shiyanova TL, Koester A, Ford AM, Micanovic R, et al (2005) FGF-21 as a novel metabolic regulator. The Journal of clinical investigation 115: 1627–1635. 15902306 10.1172/JCI23606 PMC1088017

[11] Mraz M, Bartlova M, Lacinova Z, Michalsky D, Kasalicky M, et al (2009) Serum concentrations and tissue expression of a novel endocrine regulator fibroblast growth factor-21 in patients with type 2 diabetes and obesity. Clin Endocrinol (Oxf) 71: 369–375. 19702724 10.1111/j.1365-2265.2008.03502.x

[12] Fisher FM, Chui PC, Antonellis PJ, Bina HA, Kharitonenkov A, et al (2010) Obesity is a fibroblast growth factor 21 (FGF21)-resistant state. Diabetes 59: 2781–2789. 20682689 10.2337/db10-0193 PMC2963536

[13] Tan BK, Hallschmid M, Adya R, Kern W, Lehnert H, et al (2011) Fibroblast growth factor 21 (FGF21) in human cerebrospinal fluid: relationship with plasma FGF21 and body adiposity. Diabetes 60: 2758–2762. 21926274 10.2337/db11-0672 PMC3198100

[14] Li H, Bao Y, Xu A, Pan X, Lu J, et al (2009) Serum fibroblast growth factor 21 is associated with adverse lipid profiles and gamma-glutamyltransferase but not insulin sensitivity in Chinese subjects. The Journal of clinical endocrinology and metabolism 94: 2151–2156. 19318452 10.1210/jc.2008-2331

[15] Riddell DR, Zhou H, Comery TA, Kouranova E, Lo CF, et al (2007) The LXR agonist TO901317 selectively lowers hippocampal Abeta42 and improves memory in the Tg2576 mouse model of Alzheimer's disease. Mol Cell Neurosci 34: 621–628. 17336088 10.1016/j.mcn.2007.01.011

[16] Uebanso T, Taketani Y, Yamamoto H, Amo K, Tanaka S, et al (2012) Liver X receptor negatively regulates fibroblast growth factor 21 in the fatty liver induced by cholesterol-enriched diet. J Nutr Biochem 23: 785–790. 21889884 10.1016/j.jnutbio.2011.03.023

[17] Bose AK, Mocanu MM, Carr RD, Brand CL, Yellon DM (2005) Glucagon-like peptide 1 can directly protect the heart against ischemia/reperfusion injury. Diabetes 54: 146–151. 15616022 10.2337/diabetes.54.1.146

[18] Sharma A, Singh M (2000) Effect of ethylisopropyl amiloride, a Na+ - H+ exchange inhibitor, on cardioprotective effect of ischaemic and angiotensin preconditioning. Molecular and cellular biochemistry 214: 31–38. 11195787 10.1023/a:1007167519596

[19] Rodrigo GC, Chapman RA (1991) The calcium paradox in isolated guinea-pig ventricular myocytes: effects of membrane potential and intracellular sodium. The Journal of physiology 434: 627–645. 2023134 10.1113/jphysiol.1991.sp018490 PMC1181438

[20] Mitra R, Morad M (1985) A uniform enzymatic method for dissociation of myocytes from hearts and stomachs of vertebrates. The American journal of physiology 249: H1056–1060. 2998207 10.1152/ajpheart.1985.249.5.H1056

[21] Adya R, Tan BK, Punn A, Chen J, Randeva HS (2008) Visfatin induces human endothelial VEGF and MMP-2/9 production via MAPK and PI3K/Akt signalling pathways: novel insights into visfatin-induced angiogenesis. Cardiovascular research 78: 356–365. 18093986 10.1093/cvr/cvm111

[22] Ogawa Y, Kurosu H, Yamamoto M, Nandi A, Rosenblatt KP, et al (2007) BetaKlotho is required for metabolic activity of fibroblast growth factor 21. PNAS 104: 7432–7437. 17452648 10.1073/pnas.0701600104 PMC1855074

[23] Matsui T, Li L, del Monte F, Fukui Y, Franke TF, et al (1999) Adenoviral gene transfer of activated phosphatidylinositol 3'-kinase and Akt inhibits apoptosis of hypoxic cardiomyocytes in vitro. Circulation 100: 2373–2379. 10587343 10.1161/01.cir.100.23.2373

[24] Shimizu N, Yoshiyama M, Omura T, Hanatani A, Kim S, et al (1998) Activation of mitogen-activated protein kinases and activator protein-1 in myocardial infarction in rats. Cardiovasc Res 38: 116–124. 9683913 10.1016/s0008-6363(97)00327-1

[25] Wang Y, Solt LA, Burris TP (2010) Regulation of FGF21 expression and secretion by retinoic acid receptor-related orphan receptor alpha. J Biol Chem 285: 15668–15673. 20332535 10.1074/jbc.M110.102160 PMC2871432

[26] Lionetti V, Bianchi G, Recchia FA, Ventura C (2010) Control of autocrine and paracrine myocardial signals: an emerging therapeutic strategy in heart failure. Heart Fail Rev 15: 531–542. 20364318 10.1007/s10741-010-9165-7

[27] Nanayakkara G, Kariharan T, Wang L, Zhong J, Amin R (2012) The cardio-protective signaling and mechanisms of adiponectin. Am J Cardiovasc Dis 2: 253–266. 23173099 PMC3499932

[28] Doroudgar S, Glembotski CC (2013) New concepts of endoplasmic reticulum function in the heart: programmed to conserve. J Mol Cell Cardiol 55: 85–91. 23085588 10.1016/j.yjmcc.2012.10.006 PMC3557761

[29] Attie AD, Scherer PE (2009) Adipocyte metabolism and obesity. J Lipid Res 50 Suppl: S395–399. 19017614 10.1194/jlr.R800057-JLR200 PMC2674728

[30] Planavila A, Redondo I, Hondares E, Vinciguerra M, Munts C, et al (2013) Fibroblast growth factor 21 protects against cardiac hypertrophy in mice. Nat Commun 4: 2019. 23771152 10.1038/ncomms3019

[31] Hausenloy DJ, Mocanu MM, Yellon DM (2004) Cross-talk between the survival kinases during early reperfusion: its contribution to ischemic preconditioning. Cardiovasc Res 63: 305–312. 15249188 10.1016/j.cardiores.2004.04.011

[32] Mendoza MC, Er EE, Blenis J (2011) The Ras-ERK and PI3K-mTOR pathways: cross-talk and compensation. Trends Biochem Sci 36: 320–328. 21531565 10.1016/j.tibs.2011.03.006 PMC3112285

[33] Yusuf S, Hawken S, Ounpuu S, Bautista L, Franzosi MG, et al (2005) Obesity and the risk of myocardial infarction in 27,000 participants from 52 countries: a case-control study. Lancet 366: 1640–1649. 16271645 10.1016/S0140-6736(05)67663-5

[34] Kenchaiah S, Evans JC, Levy D, Wilson PW, Benjamin EJ, et al (2002) Obesity and the risk of heart failure. N Engl J Med 347: 305–313. 12151467 10.1056/NEJMoa020245

[35] Clavijo LC, Pinto TL, Kuchulakanti PK, Torguson R, Chu WW, et al (2006) Metabolic syndrome in patients with acute myocardial infarction is associated with increased infarct size and in-hospital complications. Cardiovasc Revasc Med 7: 7–11. 16513517 10.1016/j.carrev.2005.10.007

[36] Romero-Corral A, Montori VM, Somers VK, Korinek J, Thomas RJ, et al (2006) Association of body weight with total mortality and with cardiovascular events in coronary artery disease: a systematic review of cohort studies. Lancet 368: 666–678. 16920472 10.1016/S0140-6736(06)69251-9

[37] Thim T, Bentzon JF, Kristiansen SB, Simonsen U, Andersen HL, et al (2006) Size of myocardial infarction induced by ischaemia/reperfusion is unaltered in rats with metabolic syndrome. Clin Sci (Lond) 110: 665–671. 16448385 10.1042/CS20050326

[38] Yie J, Wang W, Deng L, Tam LT, Stevens J, et al (2012) Understanding the physical interactions in the FGF21/FGFR/beta-Klotho complex: structural requirements and implications in FGF21 signaling. Chem Biol Drug Des 79: 398–410. 22248288 10.1111/j.1747-0285.2012.01325.x

[39] Jiao H, Arner P, Dickson SL, Vidal H, Mejhert N, et al (2011) Genetic association and gene expression analysis identify FGFR1 as a new susceptibility gene for human obesity. J Clin Endocrinol Metab 96: E962–966. 21430024 10.1210/jc.2010-2639

[40] Yie J, Hecht R, Patel J, Stevens J, Wang W, et al (2009) FGF21 N- and C-termini play different roles in receptor interaction and activation. FEBS Lett 583: 19–24. 19059246 10.1016/j.febslet.2008.11.023

[41] Yang C, Jin C, Li X, Wang F, McKeehan WL, et al (2012) Differential specificity of endocrine FGF19 and FGF21 to FGFR1 and FGFR4 in complex with KLB. PLoS One 7: e33870. 22442730 10.1371/journal.pone.0033870 PMC3307775

[42] Diaz-Delfin J, Hondares E, Iglesias R, Giralt M, Caelles C, et al (2012) TNF-alpha represses beta-Klotho expression and impairs FGF21 action in adipose cells: involvement of JNK1 in the FGF21 pathway. Endocrinology 153: 4238–4245. 22778214 10.1210/en.2012-1193

[43] Lopaschuk GD (1997) Alterations in fatty acid oxidation during reperfusion of the heart after myocardial ischemia. Am J Cardiol 80: 11A–16A. 9293951 10.1016/s0002-9149(97)00453-0

[44] Morabito D, Montessuit C, Rosenblatt-Velin N, Lerch R, Vallotton MB, et al (2002) Impaired glucose metabolism in the heart of obese Zucker rats after treatment with phorbol ester. Int J Obes Relat Metab Disord 26: 327–334. 11896487 10.1038/sj.ijo.0801881

[45] Bertinchant JP, Polge A, Robert E, Sabbah N, Fabbro-Peray P, et al (1999) Time-course of cardiac troponin I release from isolated perfused rat hearts during hypoxia/reoxygenation and ischemia/reperfusion. Clin Chim Acta 283: 43–56. 10404730 10.1016/s0009-8981(99)00029-7

[46] Mair J (1999) Tissue release of cardiac markers: from physiology to clinical applications. Clin Chem Lab Med 37: 1077–1084. 10726815 10.1515/CCLM.1999.157

