# Supplementary material for: Novel Insights into the Cardio-Protective Effects of FGF21 in Lean and Obese Rat Hearts
Source: PLoS One. 2014 Feb 3;9(2):e87102. doi: 10.1371/journal.pone.0087102 (PMC3911936; doi:10.1371/journal.pone.0087102)
Supplement: Figure S1 — Comparison between FGF21 and cTn-T release in Langendorff perfusates. Graphical representation of FGF21 and cTn-T (relative to basal) from Langendorff rat heart following global ischemia and reperfusion (5, 30, 60 and 120 minutes). Data shown are means ± SEM of triplicates. The values represented are relative to basal. ***P<0.001 vs. t [0] time point (FGF21), #P<0.05 vs. t [0] (cTn-T); n = 6 per group. (DOCX) [file pone.0087102.s001.docx]

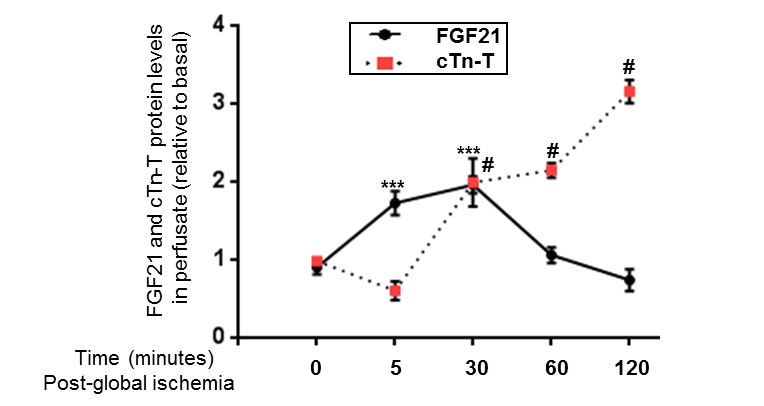


***Figure S1*.** **Comparison between FGF21 and cTn-T release in Langendorff perfusates.**

Graphical representation of FGF21 and cTn-T (relative to basal) from Langendorff rat heart following global ischemia and reperfusion (5, 30, 60 and 120 minutes). Data shown are means ± SEM of triplicates. The values represented are relative to basal. **^***^***P* < 0.001 vs. t [0] time point (FGF21), **^#^***P* < 0.05 vs. t[0] (cTn-T); n = 6 per group.
