# Supplementary material for: Novel Insights into the Cardio-Protective Effects of FGF21 in Lean and Obese Rat Hearts
Source: PLoS One. 2014 Feb 3;9(2):e87102. doi: 10.1371/journal.pone.0087102 (PMC3911936; doi:10.1371/journal.pone.0087102)
Supplement: Table S1 — Constituents of standard chow diet and high fat diet. (DOCX) [file pone.0087102.s002.docx]

**Table S1**- Constituents of standard chow diet and high fat diet.

**Standard Chow diet**

| DIET | **EURodent diet 14%** |
| --- | --- |
| INGREDIENT | % |
| Protein | 14.3 |
| Fat (ether extract)  Fat (acid hydrolysis) | 2.5  3.3 |
| Starch | 50.6 |
| Fibre (Crude) | 3.7 |
| Glucose  Fructose  Sucrose | 0.26  0.29  0.94 |
| Nitrogen-free extract (by difference) | 65.2 |
| Total digestible nutrients | 76.4 |
| Minerals (ash) | 4.1 |

**High fat diet**

| DIET | **824053 - '45% AFE Fat'** |
| --- | --- |
| INGREDIENT | g% (w/w) |
| Casein | 26.533 |
| Choline Bitartrate | 0.296 |
| L-Cystine | 0.399 |
| Lard | 17.895 |
| Rice Starch | 28.344 |
| Cellulose | 6.171 |
| Soya Oil | 4.319 |
| Sucrose | 10.490 |
| Mineral Mix | 4.319 |
| Vitamin Mix | 1.234 |
| Total | 100.000 |

| SPECIFICATION | % (w/w) | | kcal/g | | % kcal |
| --- | --- | --- | --- | --- | --- |
| Crude Fat | 22.6 | | 2.03 | | 45 |
| Crude Protein | 23.0 | | 0.92 | | 20 |
| Crude Fibre | 4.6 | | / | | / |
| Ash | 4.2 | | / | | / |
| Carbohydrate | 39.8 | | 1.59 | | 35 |
| Total AFE | | 4.54 | | 100 | |
