# Supplementary material for: Novel Insights into the Cardio-Protective Effects of FGF21 in Lean and Obese Rat Hearts
Source: PLoS One. 2014 Feb 3;9(2):e87102. doi: 10.1371/journal.pone.0087102 (PMC3911936; doi:10.1371/journal.pone.0087102)
Supplement: Table S2 — Physical and metabolic profiles in lean and high fed fat rats following 12 weeks of either standard chow diet (lean rats) or high fat diet (HF rat). (DOCX) [file pone.0087102.s003.docx]

**Table S2**. Physical and metabolic profiles in lean and high fed fat rats following 12 weeks of either standard chow diet (lean rats) or high fat diet (HF rat).

| **DIET** | **Lean rat (n=10)** | **HF rat (n=10)** |
| --- | --- | --- |
| **Age** | 18 weeks | 18 weeks |
| **Body wt, g** | 248.75+33.139 | 495+91.92^*^ |
| **Heart wt,g** | 1.1+0.09 | 1.58+0.14^**^ |
| **Insulin (pmol/l)** | 415+12 | 450+22 |
| **Glucose (mmol/l)** | 4.8+0.75 | 5.2+0.88 |
| **Cholesterol (mmol/l)** | 2.65+0.5 | 2.86+0.35 |
| **Cumulative food intake (MJ)** | 32 +2.02 | 40 +3.44^*^ |
| **Triglycerides(mmol/l)** | 1.2+0.04 | 2.1±1.0 |

The values represent the means±S.E.M. *P<0.05, **P<0.01; lean vs. high fat fed rats
