# Supplementary material for: Novel Insights into the Cardio-Protective Effects of FGF21 in Lean and Obese Rat Hearts
Source: PLoS One. 2014 Feb 3;9(2):e87102. doi: 10.1371/journal.pone.0087102 (PMC3911936; doi:10.1371/journal.pone.0087102)
Supplement: Table S3 — Primer sequences. (DOCX) [file pone.0087102.s004.docx]

**Table S3**. Primer sequences

| **Gene/productsize**  **(bp)** | **Sense primer** | **Antisense primer** |
| --- | --- | --- |
| FGF21 (189) | 5′- AGGTGACGAATGAGGGGACCACA-3′ | 5′- TCGGCTTCCCAGCCTCCAGA -3′ |
| FGFR1 (69) | 5′-TGGCACCTGAGGCATTGTT -3′ | 5′-AAGAGCACCCCAAAAGACCAC-3′ |
| βKlotho (128) | 5′-CAGAGAAGGAGGAGGTGAGG-3’ | 5′-CAGCACCTGCCTTAAGTTGA-3’ |
| GAPDH (185) | 5′- GAGTCAACGGATTTGGTCGT-3′ | 5′- GACAAGCTTCCCGTTCTCAG-3′ |

Bp-base pairs; GAPDH- *Glyceraldehyde 3-phosphate dehydrogenase* –house keeping gene.
