# Supplementary material for: Novel Insights into the Cardio-Protective Effects of FGF21 in Lean and Obese Rat Hearts
Source: PLoS One. 2014 Feb 3;9(2):e87102. doi: 10.1371/journal.pone.0087102 (PMC3911936; doi:10.1371/journal.pone.0087102)

**Table S4.** Haemodynamic parameters of isolated rat hearts. [HR-Heart Rate; AP,-Coronary flow; LVDP-Left Ventricular Developed Pressure; ±dP/dtmax. Data are means ± SEM. **P*<0.05, ***P*<0.01 vs. control.

(**A**) Pre-ischaemic


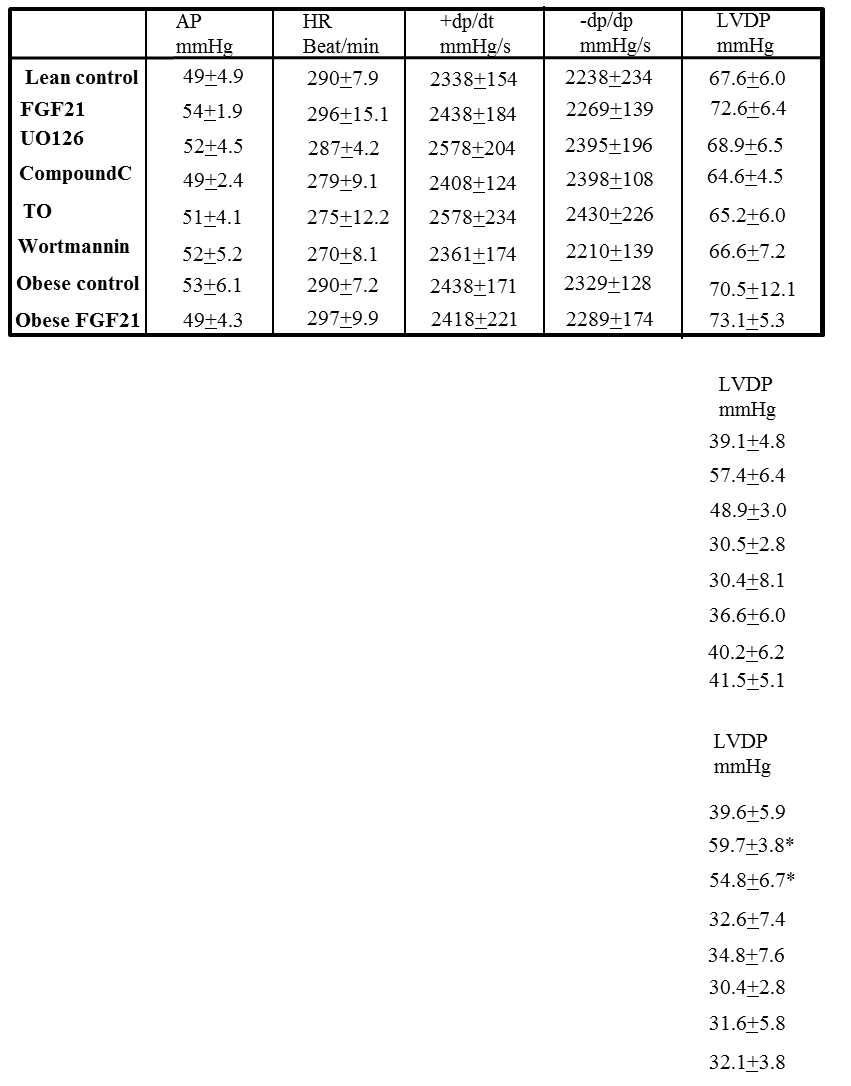


(**B**) 10min reperfusion


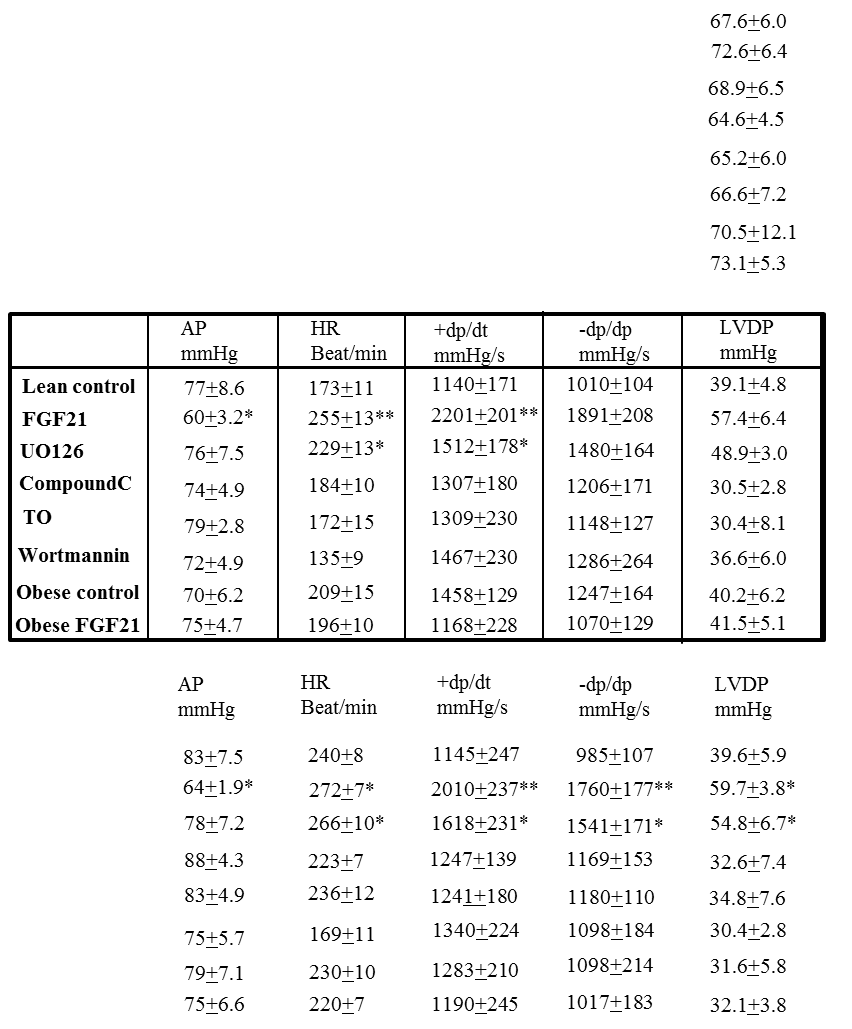


(**C**) 120min reperfusion.


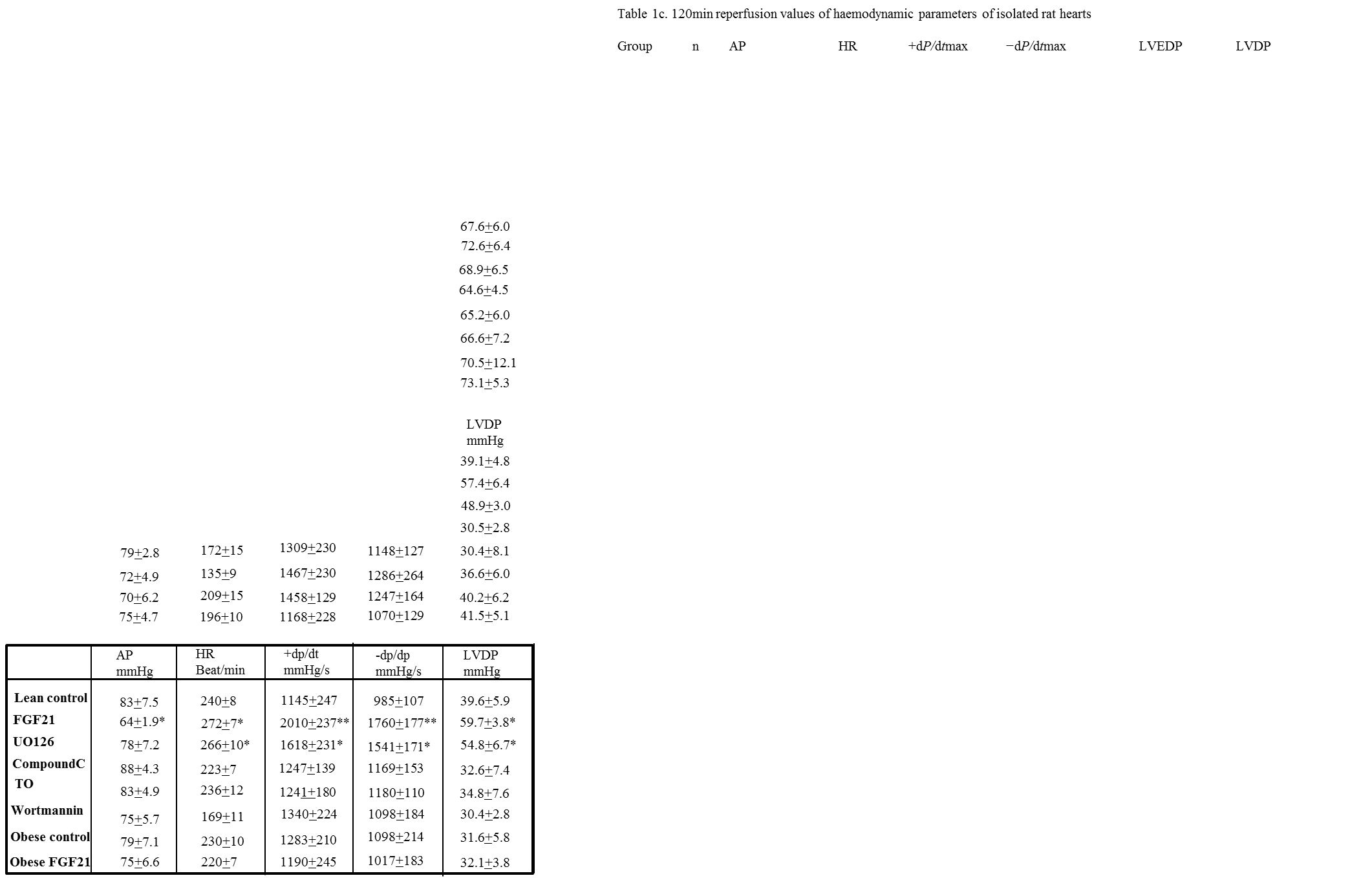

Supplement: Table S4 — Haemodynamic parameters of isolated rat hearts. (DOCX) [file pone.0087102.s005.docx]
