# Supplementary material for: Novel Insights into the Cardio-Protective Effects of FGF21 in Lean and Obese Rat Hearts
Source: PLoS One. 2014 Feb 3;9(2):e87102. doi: 10.1371/journal.pone.0087102 (PMC3911936; doi:10.1371/journal.pone.0087102)
Supplement: Supporting Information S1 — Supporting Materials and Methods, and Results. (DOC) [file pone.0087102.s006.doc]

**SUPPORTING INFORMATION**

**Materials and Methods**

***Animals***

Adult male wild-type wistar rats were obtained from Charles River labs, UK, Ltd. All rats were housed individually and maintained under pathogen-free conditions with controlled temperature and humidity and a 12-h light (0700–1900 h), 12-h dark (1900–0700 h) cycle. Following weaning, rats had free access to standard chow diet or high fat diet and water. standard diet was obtained from LabDiet., London, UK, and high-fat diet from Special Diet Services., UK. Standard diet (EURodent diet) consisted of 76.6 % carbohydrate, 6.6 % fat, and 16.8 % protein, with an energy density of 3.18 kcal/g, whereas the high-fat diet consisted of 22.6 % fat, 39.8% carbohydrate, and 23% protein, with an energy density of 4.54 kcal/g (see Table-S1). Following 12 weeks of differential diet feeding, control (lean) rats weighed 248.75+33.139 gms and high fat diet rats 495+91.92 gms respectively.

# *Isolated Langendorff Preparation*

# After a week of habituation, rats were sacrificed by intraperitoneal administration of sodium pentobarbital (200mg/kg), following which the hearts were rapidly excised, immersed in ice-cold oxygenated Tyrodes solution. The hearts were immediately perfused *via* the aortic cannula in a modified Langendorff mode with Tyrode’s solution at 37^o^C. The composition of Tyrode's solution consisted of (mM): NaCl-118, NaHCO_3_-24, CaCl_2_.2H2O-2.5, KCl-4.7, KH_2_PO_4_-1.2, MgSO_4_-7H_2_O-1.2, Glucose-11. The pH of the solution was maintained at 7.4 by continuously bubbling with 95%O2/5%CO2 and temperature maintained at 37 °C. A constant perfusion rate of 10ml.min^-1^ was maintained using a Gilson Minipuls 2 peristaltic pump (Gilson Inc., Ohio, USA). The flow was adjusted once the hearts were mounted on the Langendorff system within the first minute, flow was between 10-15ml/g wet wt/min. Adequate perfusion was established as indicated from the Aortic perfusion pressure recording, in this constant flow system. Contractile parameters were measured by insertion a fluid-filled latex balloon through the left atrium into the left ventricular, this was connected to a pressure transducer (MTL0380 Ltd, ADInstruments, UK) and the balloon volume was adjusted to give end-diastolic pressure of <10mmHg. APP was monitored with a second pressure transducer in series with the aortic cannula. Data were continuously recorded using a Power Lab 8 preamplifier/digitiser (AD Instruments UK).

*Determination of infarction following ischemia/reperfusion in the isolated Langendorff perfused rat heart*

All experiments lasted a total of 190mins. Hearts were allowed to stabilize for 30mins with the standard Tyrodes perfusion. Contractile parameters (left ventricular systolic pressure, LVSP mmHg and left ventricular end diastolic pressure, LVEDP mmHg) were measured at t0-t195 minutes. The difference between these two pressure values represented the left ventricular developed pressure (LVDP, mmHg). Heart rate (HR, beats.min^-1^) was determined at the same time together with rate pressure product (RPP, mmHg), which was calculated by multiplying LVDP with HR. Measurements recorded after a 30mins stabilisation period formed baseline values (t0mins). Global ischemia was induced for 30mins by cessation of physiological solution inflow to the heart. The hearts were maintained in a thermo-controlled chamber (37^o^C) throughout the protocol. The ischaemic period was followed by reperfusion lasting 120mins. At the conclusion of the experiment, hearts were snap frozen in liquid nitrogen. Frozen tissues were sliced into 2mm thick transverse sections and incubated in 1% triphenyl-tetrazolium chloride in phosphate buffer (pH 7.4, 37^o^C) for 15mins.

***Determination of infarct size by planimetry***

The heart slices were traced onto a clear acetate sheet and infarct areas of each section were measured by computer-assisted planimetry. The total volume from each slice of the heart was calculated by multiplying the area by 2 mm, i.e. thickness of the heart slice.

***Isolation and culture of primary adult rat cardiomyocytes***

This was then followed by perfusion with Ca^2+^-free tyrode for 5min and a further 10min perfusion with enzyme solution (Ca^2+^-free Tyrode + collagenase type I + protease type XIV) (Sigma) . Following enzymatic digestion, hearts were placed in normal Tyrodes with gentle agitation in a shaking water bath at 37°C; the isolated myocytes were then dispersed over a period of at least 30mins. Cell viability tests were performed indicating 70-80% viable yield using this method. Isolated cardiomyocytes were treated with recombinant FGF21 with or without inhibitors following dose and time optimization experiments.

***Immunohisto/cyto chemistry and confocal microscopy***

Immunofluorescence (IF): Superfrost glass slides were coated with BD cell tak adhesive according to manufacturer’s protocol [BD biosciences, UK] and air dried; following which isolated cardiomyocyte cell suspension was added in optimized densities.

The slides were then fixed with cold acetone for 10mins and washed three times with cold PBS. The cells were blocked in 1% BSA in PBST (blocking buffer) for 30mins. After washing again in PBS twice for 5mins each, the cells were incubated with mouse FG21 antibody (Abnova) diluted 1:100 in the blocking buffer for 1hr at room temperature. Washing steps were repeated and the cells were incubated with anti-mouse secondary antibody coupled to Alexa flour 488 (Life Technologies, Paisley, UK) diluted 1:200 in the blocking buffer for 1hr at room temperature in dark, washing step were repeated and cover slipped using DAPI containing mounting medium (Vector, Orton Southgate, UK). Slides were examined using a Zeiss LSM-510 confocal microscope (Zeiss, Jena, Germany) and the acquisition parameters were kept constant and below background signal (as determined by samples incubated without primary antibody).

Immunohistochemistry (IHC): IHC was performed on the formalin fixed paraffin embedded tissues using Novolink Polymer detection system (Leica, Microsystems, Milton Keynes, UK) following manufacturers’ recommendation. Heat induced antigen retrieval (HIER) was performed using pH 9 buffer (Dako, Ely, UK) and the sections were incubated overnight with FGF21 (Abnova) antibody (1:100) or PBST (negative control) overnight at 4ºC. Images were acquired using Mirax scanner (Carl Zeiss, Cambridge, UK).

***RNA isolation and real-time quantitative reverse transcription polymerase chain reaction.*** Total cellular and tissue RNA was extracted using the RNeasy Mini Kit (Qiagen Ltd., UK) and QIAGEN - RNeasy Fibrous Tissue Mini Kit, according to the manufacturer’s protocol. This was followed by reverse transcription into cDNA, by using 5 IU/ RevertAid H Minus M-MuLV Reverse Transcriptase (Fermentas, York, UK)**.** Primer sequences used for the detection of corresponding gene expression levels as listed in **Table S3**. Protocol conditions consisted of denaturation at 94 °C for 1min, then 40 cycles of 94 °C for 30s, 60 °C for 45s, and 72 °C for 30s, followed by a 7mins extension at 72 °C. Identity of the PCR products were further confirmed using sequence analyses.

***Sequence Analysis***

The PCR products from all samples were purified from the 1% agarose gel using the QIAquick Gel Extraction Kit (Qiagen). PCR products were then sequenced in an automated DNA sequences, and the sequence data were analysed using Blast nucleic acid database searches from the National Centre for Biotechnology Information, confirming the identity of our products.

***Western Blot Analysis***

Protein levels of FGF21, and its regulation, in obese and ischemic rat hearts were measured by western blot analyses. Similarly, FGF21 induced activation of signalling cascades including ERK_1/2_, Akt and AMPK in isolated rat cardiomyocytes were measured. Dose and time dependent optimisation experiments were performed with recombinant FGF21 (0-100nM) for time (0-120mins) [for signalling experiments].

Following the treatments, rat hearts were snap frozen in liquid nitrogen and stored at -70°C for later use. Briefly, protein lysates were prepared by homogenizing rat heart tissues in RIPA lysis buffer (Santa Cruz Biotechnology Inc.). Protein concentrations in the lysates were equalised using the BCA method. Eighty micrograms of each sample were separated on a 10% Sodium Dodecyl Sulfate (SDS)-polyacrylamide gel, and electro-blotted onto a polyvinylidene fluoride (PVDF) membrane (Millipore, Bedford, MA, USA). The PVDF membrane was then incubated with 5% Bovine Serum Albumin (Sigma) [BSA] in 1M Trizma/base, 1.54M NaCl, 0.05% Tween 20 (Tris buffered solution plus Tween 20, TBST, pH 7.4) for one hour at room temperature, and then exposed overnight at 4°C to TBST containing either of these primary antibodies {FGF21 antibody [(mouse monoclonal IgG, H00026291-M01, dilution 1:1000), ABCAM, UK], β-Klotho antibody [(rabbit polyclonal IgG, ab76356, dilution 1:1000), ABCAM, UK], phos ERK_1/2_ [(rabbit polyclonal IgG, 4370, dilution 1:1000), Cell Signalling, UK], phos Akt [(rabbit polyclonal IgG, 4056, dilution 1:1000), Cell Signalling, UK], phos AMPK [(rabbit polyclonal IgG, 5759, dilution 1:1000), Cell Signalling, UK}.The membranes were then washed thoroughly for 60mins with TBS/0.1% Tween before incubation with anti-rabbit/mouse secondary antibody, horseradish-peroxidase-conjugated Ig (1:5000) (Dako Ltd, Cambridge, UK) for 1hr at room temperature. Antibody complexes were visualized using chemiluminescence (ECL; GE Healthcare, Little Chalfont, UK). Band densities were measured using a scanning densitometer coupled to scanning software Scion Image™ (Scion Corporation, Maryland, U.S.A). In addition to quantitative loading of gels, membranes were also re-probed with either β-actin antibody (Cell Signalling Technology Inc., Beverly, MA, USA; 1 in 10,000 dilutions) or total ERK_1/2_/total Akt/total AMPK respectively to determine equal protein loading.

**Results**

**Determination of cardiac troponin release (in Langendorff perfusates) as a marker of cardiac death, following global ischemia and reperfusion.**

To determine the relationship between cardiac release of FGF21 and cardiac troponin-T [(cTn-T) a marker of cardiomyocyte death] [1], we measured the levels in Langendorff perfusates during ischemia and reperfusion. As depicted in **Figure S1**, the cardiac release of cTn-T was significantly increased following 30, 60 and 120 minutes of reperfusion

Interestingly, FGF21 and cTn-T release seems to follow divergent secretory patterns during reperfusion again partly supporting the cardio-protective effect of FGF21 release.

**References**

1. Bertinchant JP, Polge A, Robert E, Sabbah N, Fabbro-Peray P, et al. (1999) Time-course of cardiac troponin I release from isolated perfused rat hearts during hypoxia/reoxygenation and ischemia/reperfusion. Clin Chim Acta 283: 43-56.
